# Possible role of vitamins C and E co-administration in the prevention of testicular ischemia–reperfusion injury following surgical repair of torsion of the testis

**DOI:** 10.3389/fnut.2025.1660240

**Published:** 2025-11-03

**Authors:** Olajumoke Deborah Ogunleye, Oladele Ayobami Afolabi, Waidi Adeoye Saka, Bamidele Oluwole Olusola, Richard Adedamola Ajike, Olayemi Olutobi Oladokun, Sodiq Opeyemi Hammed, Oluwaseun Samuel Hezekiah, Oreoluwa Janet Adedeji

**Affiliations:** ^1^Physiology Unit, Department of Nursing Sciences, McPherson University, Abeokuta, Nigeria; ^2^Department of Physiology, Ladoke Akintola University of Technology, Ogbomosho, Nigeria; ^3^Department of Public Health, College of Health Sciences and Public Policy, Walden University, Minneapolis, MN, United States; ^4^Department of Physiology, Osun State University, Osogbo, Nigeria

**Keywords:** torsion of the testis, surgical detorsion, testicular ischemia reperfusion injury, oxidative stress, inflammation and synergistic antioxidants

## Abstract

Torsion of the testis (TT) is a recognised urological emergency whereby twisting of the spermatic cord causes testicular ischemia. Surgical detorsion restores perfusion, but inevitably results in testicular ischemia–reperfusion injury (tIRI). The resultant of this is oxidative stress, inflammation, impaired steroidogenesis, and a loss of spermatogenic function. Although single-agent antioxidants have been evaluated by previous studies in the mitigation of tIRI, there is limited evidence that addresses the additive or synergistic protection of co-administration of vitamins C and E after detorsion. Mechanistic data indicate possible complementary actions of vitamin E’s (α-tocopherol) protection of membrane lipids from peroxidation and vitamin C’s (ascorbate) clearance of aqueous reactive oxygen species, which regenerates oxidized α-tocopherol back to its active form. Together, they reduce lipid peroxidation markers, attenuate neutrophil-mediated oxidative bursts, suppress NF-κB-driven pro-inflammatory signalling, and may activate cytoprotective pathways such as Nrf2/HO-1. Preclinical studies show more consistent reductions in oxidative damage and inflammatory markers with combined treatment than with either vitamin alone. However, most evidence derives from animal and *in vitro* models. Hence, heterogeneity in dosing, timing (pre- vs. post-treatment), and outcome measures limits direct clinical translation. This review, therefore, examines preclinical and mechanistic studies of vitamins C and E co-administration in models of tIRI and related ischemic injuries.

## 1 Introduction

Torsion of the testis (TT) is one of the urological emergencies seen in neonatal or adolescent males that requires early diagnosis and treatment to prevent testicular loss and preserve future fertility (1). TT is described as the twisting of the testis around its spermatic cord, causing an interruption in the blood flow to and away from the testis (2). Cessation of arterial blood flow leads to hypoxia and venous congestion, which in most cases result in pain, swelling, erythema, inflammation, loss of cremasteric reflex, and premature testicular death (3). About one fourth of patients with TT may develop testicular atrophy and infertility even after surgical detorsion (repair of the twisted testis) (4). Based on an epidemiological study conducted in Nigeria, testicular torsion contributes about 5.8% to male infertility (5). Experimental models have noted that 720° torsion sustained for several hours can cause impaired spermatogenesis and lead to the irreversible loss of germ cells, particularly if detorsion is delayed beyond 4–6 h (6–8). In essence, viability is highest within 6 h and declines thereafter. However, it should be noted that viability is not uniformly lost beyond 6–24 h, thereby reinforcing the need for urgent action without the implication of futility after 6 h (9). While the restoration of blood flow at detorsion protects the ischemic testis against necrosis, it inevitably precipitates testicular ischemia–reperfusion injury (tIRI) (1, 10, 11). Reperfusion triggers a burst of reactive oxygen species (ROS), endothelial dysfunction, neutrophil recruitment, and inflammatory signalling (e.g., NF-κB), which leads to the disruption of endogenous cytoprotective pathways (e.g., Nrf2/HO-1), as well as the depletion of antioxidants, including superoxide dismutase (SOD) and catalase, hence culminating in germ-cell loss and impaired Sertoli and Leydig cell function (12). Also, the degree of twist and duration jointly determine injury severity (higher degrees, such as 720°, causing more rapid ischemia), but even with technically successful detorsion, IRI can drive atrophy and subfertility.

In the testis, there is a need to create a balance between the reactive oxygen species (ROS) generated and antioxidant system in order to protect it against tIRI (13). The body’s antioxidant defence system are capable of mopping up ROS generated during metabolic processes. However, these antioxidants are usually depleted in pathological conditions, including tIRI (14). Upon depletion, ROS damages the cytoarchitecture, and predisposes the cell to a cascade of events that culminate in death. Particularly, in tIRI, redox imbalance is a key pathological event that contributes to the positive feedback loop of damage in the testis (15, 16). Interestingly, the body’s antioxidant system can be preserved/upregulated via the intake of exogenous antioxidants, which are capable of scavenging ROS directly in order to prevent oxidative stress-induced IRI. Therefore, it is plausible that the use of antioxidant vitamins may be used to boost the body’s antioxidant system in order to mitigate damage from oxidative stress-induced processes (17, 18).

Some dietary vitamins can prevent the harmful effects of ROS on the testis via non-enzymatic pathways like vitamin C (ascorbic acid/ascorbate) and vitamin E (α-tocopherol). These vitamins have been used in several in-vitro experimental studies and reviews (19–21). Vitamin C is a water-soluble antioxidant considered as ‘the forefront defense’ against aqueous free radicals through ROS neutralization, reduction of peroxides, repair of peroxidized cell membranes and sequestration of iron (22). Vitamin C provides high-energy electrons via oxidation to neutralize free aqueous radicals causing them to regain their stability and quench their reactivity (23). On the other hand, vitamin E is a lipophilic antioxidant, and is widely used due to its action against peroxidation reactions in cell membranes by neutralizing lipid peroxyl radicals (LPO•) (24). Vitamin E breaks the lipid peroxyl radical chain reactions by donating hydrogen atom via oxidation and averting the peroxidation of membrane lipids (25). Importantly, ascorbate can regenerate α-tocopherol from the tocopheroxyl radical, hence providing a mechanistic basis for combination therapy (20). Several studies have reported this synergistic action between these vitamins when combined (19, 20, 26). Also, while many studies have used vitamins C and E, both separately and combined, to prevent chemical or toxicant-induced testicular injury (19, 20, 27, 28), there has not been any study reporting the combined use of these vitamins to prevent tIRI.

This is partly because the translational feasibility of these agents depends on their pharmacokinetics and timing in emergency settings. Oral vitamin C exhibits saturable transport with plasma levels, usually plateauing near ~70–80 μM. Intravenous dosing has been noted to transiently achieve much higher concentrations and has been used peri-procedurally in IRI contexts (29). On the other hand, α-Tocopherol absorption requires chylomicron transport, which explains the slower rises in plasma/tissue levels, and consequently suggests peri-operative “rapid loading” is more practical for vitamin C than for vitamin E (30). Hence, vitamin E may be better suited for early post-operative dosing to support membrane protection during ongoing inflammatory/oxidative signalling. Furthermore, safety considerations, such as high-dose IV vitamin C in renal risk, and vitamin E–associated bleeding risk, should also be considered (31). Additionally, because most rodent models endogenously synthesize vitamin C, this can complicate dose translation to humans (30).

## 2 Torsion of the testis (TT)

**Figure 1:**
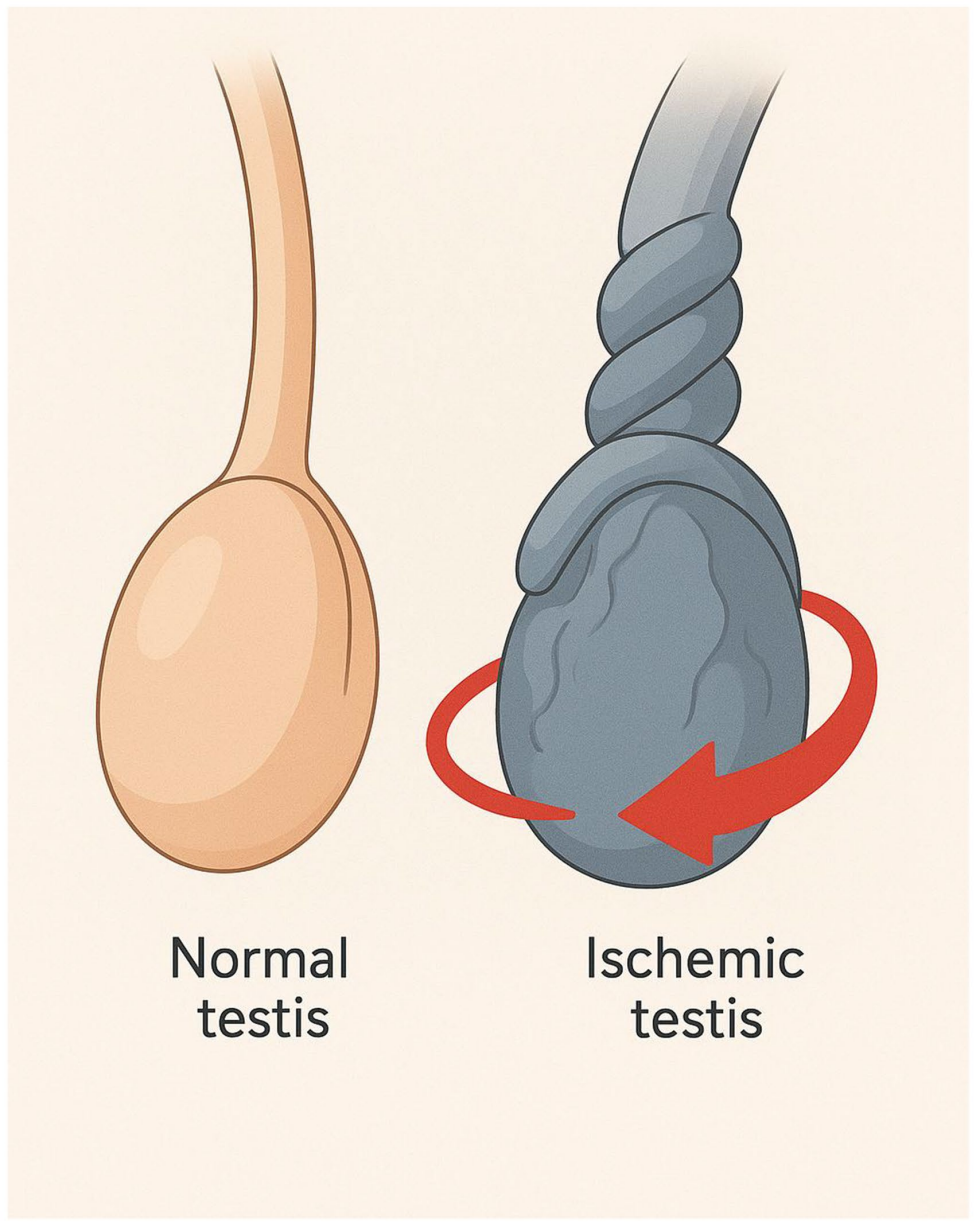
Schematic illustration of testicular torsion. The figure illustrates the anatomical comparison between a normal testis and a torsed (ischemic) testis. On the left, the normal testis appears intact with a straight spermatic cord, indicating normal blood flow. On the right, the ischemic testis shows a twisted spermatic cord.

Torsion of the testis (TT) requires early diagnosis and treatment to prevent testicular loss and preserve future fertility (1). The twisting of the testis around its spermatic cord (Figure 1) causes an interruption in the blood supply to that testis (2). When arterial blood flow is interrupted, hypoxia and venous congestion occur, causing scrotal pain, swelling, erythema, inflammation and absence of cremasteric reflex (3). One in every four thousand men under the age of twenty-five has reportedly had TT (32). Although the greatest incidence of TT is observed in the teenage age range, when the testes quickly grow in size due to a sudden rise in the levels of testosterone; however, TT is not precluded in adults or elderly individuals (33). TT has no known cause, although there are several anatomical characteristics that increase the risk, including a history of cryptorchidism, clapper bell deformity, a congenital abnormality, and a spermatic cord with a lengthy intrascrotal segment (34). Recently, excessively long-distance cycling has been reported by Coguplugil and Bedir (35), to seldomly result in testicular torsion in adults.

Surgical detorsion (SD) is done to restore blood flow (reperfusion) to an ischemic testis in order to terminate ischemic pain, hypoxia and necrosis (36). However, a number of investigations have shown that certain metabolic processes follow the restoration of blood flow, resulting in additional injury called ‘testicular ischemia–reperfusion injury-tIRI’ (12, 37). It must be emphasized that tissue damage results from both the ischemia and reperfusion phases. Most studies agree that permanent alterations start after 6 h, or even 4 h if the spermatic cord is highly twisted (38). Therefore, in the repair of TT, time is the most important issue, largely determining the salvage rate and late result (4, 38). Afolabi et al. (6) reported that a 720^°^ torsion for 1 h followed by reperfusion for 48 h is needed to cause tIRI. Delay in the treatment can lead to atrophy of the ipsilateral testis and suppression of the contralateral testis’ functions, having a variable effect on reproduction. Therefore, delayed SD can be fatal and may lead to impaired fertility and loss of the testicles (39).

## 3 Complications associated with the surgical repair of torsion of the testis

### 3.1 Testicular ischemia reperfusion injury (tIRI)

**Figure 2:**
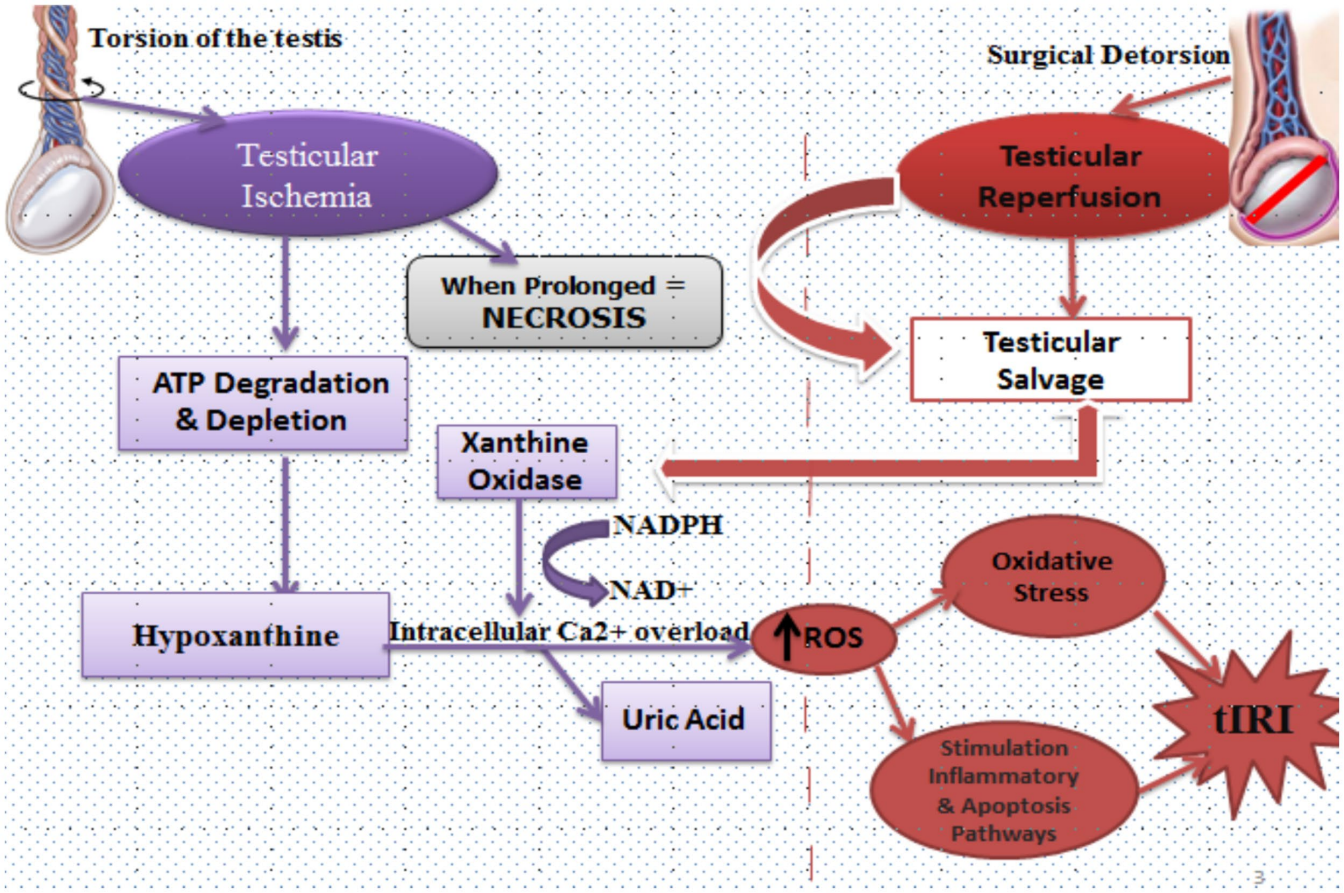
Mechanisms of testicular ischemia reperfusion injury (tIRI). During testicular torsion, arterial occlusion leads to ischemia characterized by ATP degradation and depletion. This metabolic stress drives conversion of ATP to hypoxanthine. Under hypoxic conditions, xanthine dehydrogenase is converted to xanthine oxidase. On reperfusion, the re-introduction of oxygen allows xanthine oxidase to metabolize hypoxanthine to uric acid, generating reactive oxygen species (ROS) in the process. Simultaneously, ischemia impairs ionic homeostasis, causing intracellular Ca2+ overload and NADPH/NAD+ imbalance, further amplifying ROS generation. Following surgical detorsion, reperfusion salvages ischemic tissue but also triggers a surge in ROS that overwhelms endogenous antioxidants. This initiates lipid peroxidation, mitochondrial dysfunction, and activation of inflammatory and apoptotic pathways. If the ischemic duration is prolonged, necrosis ensues and testicular salvage becomes unlikely. Together, these processes define tIRI as the paradoxical injury caused by restoring blood flow after torsion.

Testicular injury that results from restoring blood flow to an ischemic testis is called testicular-ischemia–reperfusion injury (tIRI), often referred to as testicular re-oxygenation injury (40). Cessation of blood flow to the testis (TT) induces hypoxia and premature testicular loss, and while reperfusion preserves ischemic testis, it also sets off a paradoxical chain of events leading to tIRI, as shown in Figure 2 (41, 42). During testicular ischemia, degradation of ATP to adenosine diphosphate (ADP), then to adenosine monophosphate (AMP), to adenosine, and finally to hypoxanthine occurs via anaerobic glycolysis (43, 44).

Under lower ATP conditions, the enzyme xanthine dehydrogenase undergoes a conformational change to xanthine oxidase, which, on reperfusion, triggers the formation and release of free radicals (superoxide and hydrogen peroxide) during the degradation of hypoxanthine to uric acid (45, 46). During ischemia, the membrane ion pumps, including Na^+^/K^+^-ATPase and Ca^2+^-ATPase, are also disrupted, consequently leading to intracellular acidosis and activation of the Na^+^/H^+^ exchanger. As a result, there is an accumulation of H^+^ ions in the cytosol, which causes a fall in the intracellular pH. To maintain normal intracellular pH, the cell forces out H^+^ ions in exchange for Na^+^ ions via the Na^+^/H^+^ exchanger system; likewise, Na^+^ ions are also swapped for Ca^2+^ ions by the plasmalemmal Na^+^/ Ca^2+^ exchanger, resulting in intracellular Ca^2+^ overload (47). The secondary reverse-mode Na^+^/Ca^2+^ exchange further promotes cytosolic Ca^2+^ overload (47, 48).

In the early reperfusion phase, the mitochondria take up the accumulated Ca^2+^. Ca^2+^ overload together with ROS favours the opening of the mitochondrial permeability transition pore (mPTP) and outer-membrane permeabilization, thereby facilitating the release of cytochrome c and SMAC/DIABLO from mitochondria into the cytosol to drive caspase activation and apoptosis (46, 49–53). Ca^2+^ elevation can also activate calpains, which are a cysteine protease family, capable of degrading cytoskeletal and organellar proteins (37). In parallel, Ca^2+^ overload and ROS can prime/activate inflammasome signalling, thereby amplifying cytokine release (52).

While re-oxygenation is required to restore aerobic metabolism and save the ischemic tissue, the major burst of ROS occurs on reperfusion rather than during ischemia (37, 42). With increased intracellular calcium and consumption of nicotinamide adenine dinucleotide (NAD^+^), XO-dependent purine catabolism further contributes to ROS formation due to the conversion of hypoxanthine to uric acid (45). Key ROS generated during reperfusion include superoxide (O₂•^−^), hydrogen peroxide (H₂O₂), and the hydroxyl radical (•OH) (49). When ROS generation exceeds antioxidant capacity, oxidative stress ensues, which damages proteins, lipids, and DNA, as well as activates apoptotic machinery, which usually goes beyond the initial insult of the ischemic phase (12, 37, 51, 54).

Generally, the primary clinical context where tIRI is indicated is torsion followed by detorsion/surgical repair. However, testicular transplantation can offer a parallel surgical scenario, having similar pathophysiological implications. Barten and Newling (55) and Stanley (56) traced the controversial history of testicular transplantation from surgical attempts by early pioneers to controlled animal studies. Contemporary transplantation of testicular tissue, or whole-organ grafting, as it is called, now primarily occurs within the context of fertility preservation and experimental revascularized graft models rather than “glandular rejuvenation,” which marked early endeavours by clinicians (57, 58). Although full spermatogenic recovery remains inconsistent, autologous grafting and re-implantation of cryopreserved immature or adult testicular tissue have reported survival of graft fragments and re-establishment of somatic cell markers (59). In essence, testicular transplantation is feasible but constrained by ischemic intervals during retrieval, cold storage or revascularization, as well as host–graft interactions (58, 60).

During transplantation, the grafted testicular tissue is inevitably subjected to a period of ischemia (either cold preservation or loss of perfusion), followed by reperfusion once vascular connections are restored. This process can provoke oxidative stress, inflammatory responses, apoptosis, and impairment of spermatogenesis analogous to that of post-detorsion I/R in torsion (61, 62).

### 3.2 Oxidative stress and antioxidant defense

Oxidative stress is a condition of imbalance between the production of free radicals and the biological system’s ability to quickly detoxify reactive mediators or quickly repair the damage caused (63, 64). Oxidative stress can affect every component of the testes, including the germ cells, spermatozoa, Sertoli cells, Leydig cells, and seminiferous tubules (65). This imbalance is a central pathogenic mechanism in testicular ischemia–reperfusion injury (tIRI). Major sources of ROS during reperfusion include mitochondrial electron transport chain dysfunction, xanthine oxidase activation, NADPH oxidase, and uncoupled nitric oxide synthase (63–65). Also, the main species implicated are superoxide (O₂•^−^), hydrogen peroxide (H₂O₂), hydroxyl radical (•OH), and peroxynitrite (ONOO^−^) (66).

Free radicals are very unstable and reactive with other compounds due to unpaired electrons in their outermost shell. Reactive oxygen species, or reactive oxygen molecules, are created when an oxygen molecule (O2) experiences a four-electron reduction upon reperfusion (66). Because of their archly reactive character, ROS can readily combine with different molecules, directly causing oxidation that can result in structural and functional alterations and cell damage (67). At the molecular level, these oxidants attack lipids, proteins, and nucleic acids, producing lipid peroxidation products such as malondialdehyde (MDA) and 4-hydroxynonenal, oxidized DNA bases like 8-OHdG, and protein carbonyl derivatives (67). These by-products are usually used as biomarkers of oxidative damage in experimental torsion–detorsion models. Elevated MDA and depleted reduced glutathione (GSH) have consistently been reported in ischemic testes (68, 69).

Lipid peroxidation, usually assessed by MDA, is a chain reaction in which unsaturated fatty acids (components of cell membranes) are oxidized to produce free radicals such as hydroxyl radical (HO·), hydroperoxyl radical (HOO·), lipid peroxyl radical (LOO·), and alkoxyl radicals (LO·). The peroxidation chain reaction will propagate once it has started (70). The LPO radicals destroy testicular macromolecules and induce cytotoxic, genotoxic and inflammatory reactions (71). Lipid peroxidation is a consequential factor that causes localized damage to seminiferous tubules and alters the activity of membrane-bound steroidogenic enzymes and receptors (72). Among the agents that protect the testes from lipid peroxidation, vitamin E (lipid soluble) is considered the most important (73). The abilities of this vitamin to scavenge lipid peroxyl radicals and so stop the propagation of free radical chain reactions have drawn attention to it as an exogenous antioxidant (74).

The biological relevance of oxidative stress in TT is supported by experimental interventions. For instance, GSH supplementation significantly improved post-thaw sperm function and reduced oxidative stress markers in avian reproductive models (75). This demonstrates that restoring intracellular redox buffering is essential for sperm preservation and, by extension, relevant to tIRI (65).

### 3.3 Activation of inflammatory pathways

Reperfusion causes many changes in endothelial cells such as increased membrane permeability and recruitment of inflammatory cells (48). Inflammation is a critical contributor to tIRI and often acts synergistically with oxidative stress. At the molecular level, ROS and Ca^2+^ overload trigger redox-sensitive transcription factors, particularly nuclear factor kappa B (NF-κB). NF-κB activation induces the expression of pro-inflammatory cytokines, including tumour necrosis factor-α (TNF-α), interleukin-1β (IL-1β), and interleukin-6 (IL-6), as well as downstream inflammatory mediators such as inducible nitric oxide synthase (iNOS) (76). These cytokines perpetuate germ cell apoptosis, impair Leydig cell steroidogenesis, and compromise the blood–testis barrier, thereby exacerbating subfertility risk. Complement system activating products (anaphylatoxins-C5a and membrane attack complex C5b-9) further induce inflammatory effects such as neutrophil chemotaxis, protease release, and O_2_ radical production, all of which additionally increase the response of neutrophil chemotaxis (77, 78).

The release of inflammatory cytokines and inflammatory genes (STAT3, CCR1, RAC1, MMP9, CCR10, CSF3R and HTRA1) has been noted to be stimulated by myeloperoxidase (MPO) and nuclear factor kappa B (NF-κB), respectively to initiate inflammatory reactions (79, 80).

Experimental models confirm the relevance of inflammation to testicular injury. The pharmacological inhibition of NF-κB, genetic ablation of MPO, or cytokine blockade each attenuates histological damage and reduces apoptosis after torsion–detorsion (79). In support of this, natural antioxidants with anti-inflammatory activity, such as hydro-alcoholic extract of *Quercus brantii*, were shown to downregulate NF-κB activation and lower oxidative/inflammatory markers in male reproductive tissues exposed to lead toxicity (80). Despite consistent findings from animal models, meta-analyses or systematic syntheses specific to TT are lacking. Most available data are experimental and heterogeneous in design, that is, species, torsion duration, and pharmacological interventions, all of which limit quantitative comparison. Nonetheless, the convergence of evidence supports inflammation as a mechanistic driver of tIRI alongside oxidative stress, reinforcing the rationale for combination antioxidant therapies that also possess anti-inflammatory potential.

## 4 Therapeutic strategy to prevent testicular ischemia reperfusion injury (tIRI): role of vitamins C and E

Non-enzymatic antioxidants are also known as exogenous antioxidants. These antioxidants have the capacity to scavenge ROS produced in the testicular tissues to prevent peroxidation of plasma membrane lipids (17, 81). They are usually consumed with food and include vitamins C and E (20).

### 4.1 Vitamin C (L-ascorbic acid) and its role in protecting testicular function

**Figure 3:**
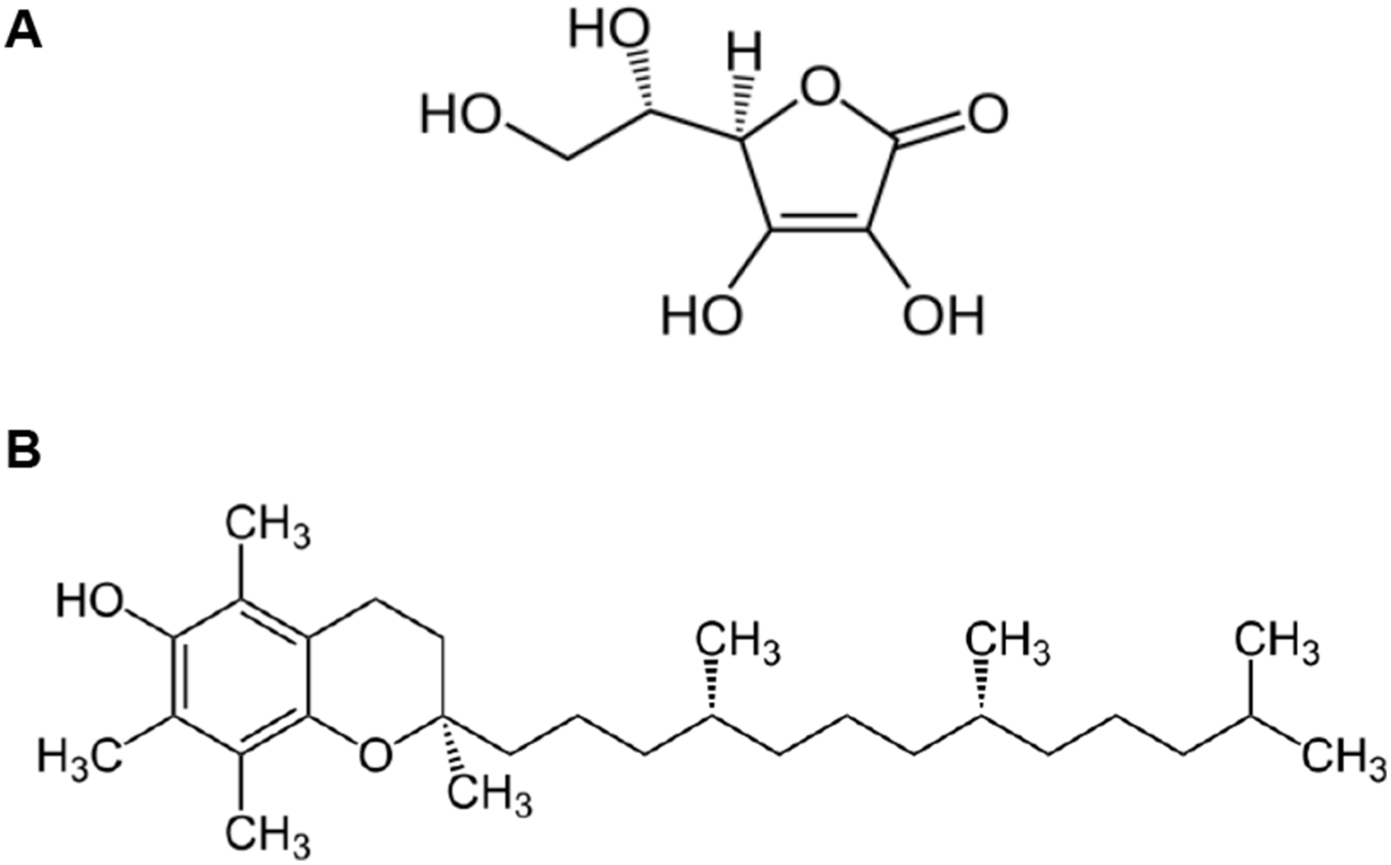
Chemical structure of Vitamin C **(A)** and Vitamin E **(B)**. **(A)** Vitamin C (L-Ascorbic Acid): The structure shows a six-membered lactone ring with multiple hydroxyl (-OH) groups at positions 2, 3, 5, and 6, and a characteristic enediol group (C=C with adjacent OH groups) between carbons 2 and 3. This water-soluble vitamin contains a primary alcohol group (-CH2OH) at carbon 6 and exhibits strong reducing properties due to its enediol moiety. The planar ring structure with its conjugated system of double bonds and hydroxyl groups enables vitamin C to readily donate electrons, making it a powerful antioxidant capable of neutralizing reactive oxygen species and regenerating other antioxidants like vitamin E. Image reproduced from the Public domain, via Wikimedia Commons. **(B)** Vitamin E (α-Tocopherol): The structure displays a complex fat-soluble molecule consisting of a chromanol head group connected to a long phytyl side chain. The chromanol ring system contains a phenolic hydroxyl group that serves as the active antioxidant site, while three methyl substituents on the ring enhance its stability. The 16-carbon saturated phytyl tail, with its characteristic methyl branches at carbons 4, 8, and 12, anchors the molecule within cell membranes and determines its lipophilic character. This structural arrangement allows vitamin E to protect membrane lipids from peroxidation by breaking free radical chain reactions. Image reproduced from the Public domain, via Wikimedia Commons.

Vitamin C (L-ascorbic acid) is a hydrophilic, non-enzymatic antioxidant that donates electrons to neutralize aqueous reactive oxygen species (ROS) and repair oxidized biomolecules, thereby limiting chain-propagating reactions in ischemia–reperfusion injury (IRI) (22). Its chemical formula is C_6_H_8_O_6_ (Figure 3A), which is made up of six carbons, eight hydrogen atoms, and six oxygen atoms (82). This structure facilitates its role as an electron donor, enabling it to neutralize a wide spectrum of reactive oxygen species (ROS), including singlet oxygen, hydrogen peroxide (H₂O₂), and the highly damaging hydroxyl radical (22, 83). In addition to direct radical scavenging, ascorbate can reduce lipid-phase radicals indirectly by regenerating α-tocopherol (vitamin E) from its tocopheroxyl radical at the aqueous–lipid interface. Its synergism with vitamin E is well-described and supports membrane integrity during oxidative bursts (82–85). This “front-line” role has been demonstrated across reproductive models; for example, vitamin C lowered oxidative markers and improved tissue integrity in cyclophosphamide-injured ovaries in mice, consistent with its capacity to sequester redox-active metals and reduce peroxides (rather than “repair” membranes directly) (86, 87).

Mechanistically, ascorbate is oxidized to semidehydroascorbate and dehydroascorbate (DHA) during radical scavenging. DHA is then recycled back to ascorbate by glutathione-dependent dehydroascorbate reductase (using GSH as the electron donor) and by NAD(P)H-dependent semidehydroascorbate reductases. These recycling steps preserve intracellular ascorbate pools and sustain α-tocopherol regeneration during reperfusion (88–90). Ascorbate also attenuates oxidative cascades that follow xanthine oxidase (XO)–driven superoxide generation after ischemia, primarily by lowering the overall oxidant burden and preserving endogenous antioxidant enzyme activities. In essence, vitamin C more accurately modulates the redox milieu than directly controlling XO conformational switching (53, 82–85, 91, 92).

In the testis, vitamin C is abundant in the seminal plasma where it helps maintain sperm DNA integrity and limits lipid peroxidation of the polyunsaturated fatty acid–rich sperm membrane (93–95). Several animal studies report improved spermatogenic indices and steroidogenic markers after ascorbate administration in oxidative injury models, though controlled human data specific to testicular torsion (TT) and tIRI remain absent (96–100). Complementary findings from cryobiology further illustrate ascorbate-like antioxidant effects in reproductive cells; for example, κ-carrageenan and fullerene (C60HyFn) additives reduced post-thaw oxidative damage in buffalo bull semen, highlighting that bolstering extracellular antioxidant capacity can preserve sperm function under oxidative stress (101). While not a TT model, the directionality is consistent with the proposed mechanism.

Rodent torsion–detorsion studies indicate that antioxidants administered before or at detorsion can curb subsequent tIRI by reducing malondialdehyde (MDA), restoring superoxide dismutase (SOD)/catalase/glutathione peroxidase (GPx) activities, and improving histology (82–85, 96–100). Vitamin C has been used alone and sometimes alongside other agents in these models. Typical doses cluster around 50–200 mg/kg i.p./i.v. given 15–60 min pre-detorsion, with some studies extending dosing into the early reperfusion window (102–104). Extrapolation to humans requires caution due to species differences in ascorbate transporters (SVCT1/2), redox enzyme expression, and the unique kinetic constraints of emergency TT care (11).

Human pharmacokinetics show that oral vitamin C displays tight control with saturable absorption. Usually, steady-state plasma concentrations plateau at ~70–80 μM at ~200–400 mg/day, whereas higher oral doses chiefly increase urinary excretion (87, 92). In contrast, intravenous vitamin C transiently achieves millimolar plasma levels that may be relevant for short, high-oxidant states like reperfusion. If vitamin C is ever tested peri-detorsion, an IV bolus at induction and a second dose shortly after reperfusion would be a rational design, paired with rigorous safety monitoring [this is a proposal, and not the current standard of care; (87, 92, 104)].

At typical dietary intakes, vitamin C is safe. For adults, the Tolerable Upper Intake Level (UL) is 2,000 mg/day orally, above which gastrointestinal upset and osmotic diarrhea occur (87). High-dose vitamin C can increase urinary oxalate, and individuals with a history of calcium oxalate nephrolithiasis or renal impairment should exercise caution for usage. G6PD deficiency is also a concern for very high-dose IV regimens due to rare hemolysis reports. Hence, this must be excluded in any interventional study. Vitamin C may also affect certain lab measurements (e.g., point-of-care glucose). However, none of these issues preclude research in TT/tIRI, but they highlight the need for defined dosing windows, monitoring of renal function/urinalysis, and exclusion criteria (87, 92, 104).

The claims of synergy with vitamin E are mechanistically sound (ascorbate regenerates α-tocopherol) and supported by *in vitro* and *in vivo* oxidative-injury models apart from TT (91). However, head-to-head TT data comparing vitamin C alone vs. C + E vs. other combinations (e.g., N-acetylcysteine, melatonin, quercetin, CoQ10) are not available. The broader torsion literature shows benefit from several antioxidants (11), but potency, timing, and tissue distribution differ, which are key variables that a future clinical trial would need to harmonize (91).

### 4.2 Vitamin E (α-tocopherol) and its role in protecting testicular function

Vitamin E, also known as α-tocopherol, is an antioxidant and lipid-soluble vitamin having the chemical formula C_29_H_50_O_2_ (Figure 3B). Generally, vitamin E refers to a family of eight fat-soluble molecules (four tocopherols and four tocotrienols), of which α-tocopherol is the most biologically active form in humans due to preferential hepatic binding to α-tocopherol transfer protein (105). Like other vitamins, vitamin E may be obtained from food, such as nuts, seeds, and vegetable oils, but it can also be taken as a supplement (106).

Vitamin E protects the plasma membrane by scavenging lipid peroxyl radicals and stopping chain reactions of lipid peroxidation in the lipid layers of the membranes (79). Its lipophilic structure allows it to insert itself into polyunsaturated fatty acid (PUFA)-rich membranes where it acts as a chain-breaking antioxidant, intercepting lipid peroxyl radicals (LOO•) and donating a hydrogen atom to terminate lipid peroxidation (107, 108). This prevents the propagation of membrane damage that is characteristic of reperfusion injury, particularly relevant in the PUFA-dense membranes of spermatozoa and germ cells (109). When α-tocopherol donates a hydrogen atom, it becomes the tocopheroxyl radical. Unlike many other antioxidants, this radical is relatively stable and can be recycled back to its active form by vitamin C or other reducing agents (110, 111). The continuous maintenance of the steady-state or low concentration of vitamin E radicals via vitamin E recycling prevents the loss or consumption of vitamin E (111). Therefore, a more significant therapeutic effect of α-tocopherol probably requires co-antioxidants such as vitamin C to have a beneficial effect.

In addition to preventing lipid peroxidation, vitamin E can also influence the redox-sensitive enzymes and transcription factors in the body. In experimental systems, α-tocopherol downregulates NADPH oxidase activity and reduces XO-mediated ROS formation indirectly by maintaining redox balance, thereby limiting the conversion of xanthine dehydrogenase to xanthine oxidase (80, 112, 113). This effect contributes to lower ROS burden during reperfusion and protects against apoptosis. Vitamin E has also been reported to stabilize mitochondrial membranes, limit cytochrome c release, and modulate Bcl-2/Bax signaling, thereby directly influencing the apoptotic threshold (80).

Although vitamin E is highly effective against lipid peroxidation, it is less potent in scavenging aqueous ROS than vitamin C or glutathione. This makes it particularly valuable in membrane-rich tissues like the testis, but less comprehensive as a stand-alone therapy. Other antioxidants such as melatonin, quercetin, and N-acetylcysteine have also demonstrated benefit in tIRI by targeting multiple redox and inflammatory pathways (114–116). Thus, vitamin E should be considered part of a broader antioxidant strategy rather than a single “magic bullet.”

Animal studies commonly use 100–200 mg/kg doses of vitamin E administered intraperitoneally or orally before detorsion (117, 118). Human physiology, however, differs significantly as oral absorption of vitamin E depends on chylomicron assembly and bile salts, making absorption variable and slower than vitamin C. On average, plasma α-tocopherol concentrations plateau at ~30 μM with typical dietary intakes, while pharmacologic supplementation (≥400 IU/day) can raise levels modestly, but this requires chronic dosing (119). High-dose vitamin E supplementation in humans has also raised safety concerns. While generally well tolerated, doses above 800 IU/day have been associated with increased risk of hemorrhagic stroke and impaired platelet aggregation due to vitamin E’s anti-vitamin K activity (120). Meta-analyses also suggest possible associations with increased all-cause mortality at very high doses, though causality remains debated. Thus, perioperative or acute IV regimens for TT/tIRI have not been established, and translation from animal models to clinical settings must carefully balance efficacy with safety.

**Figure 4:**
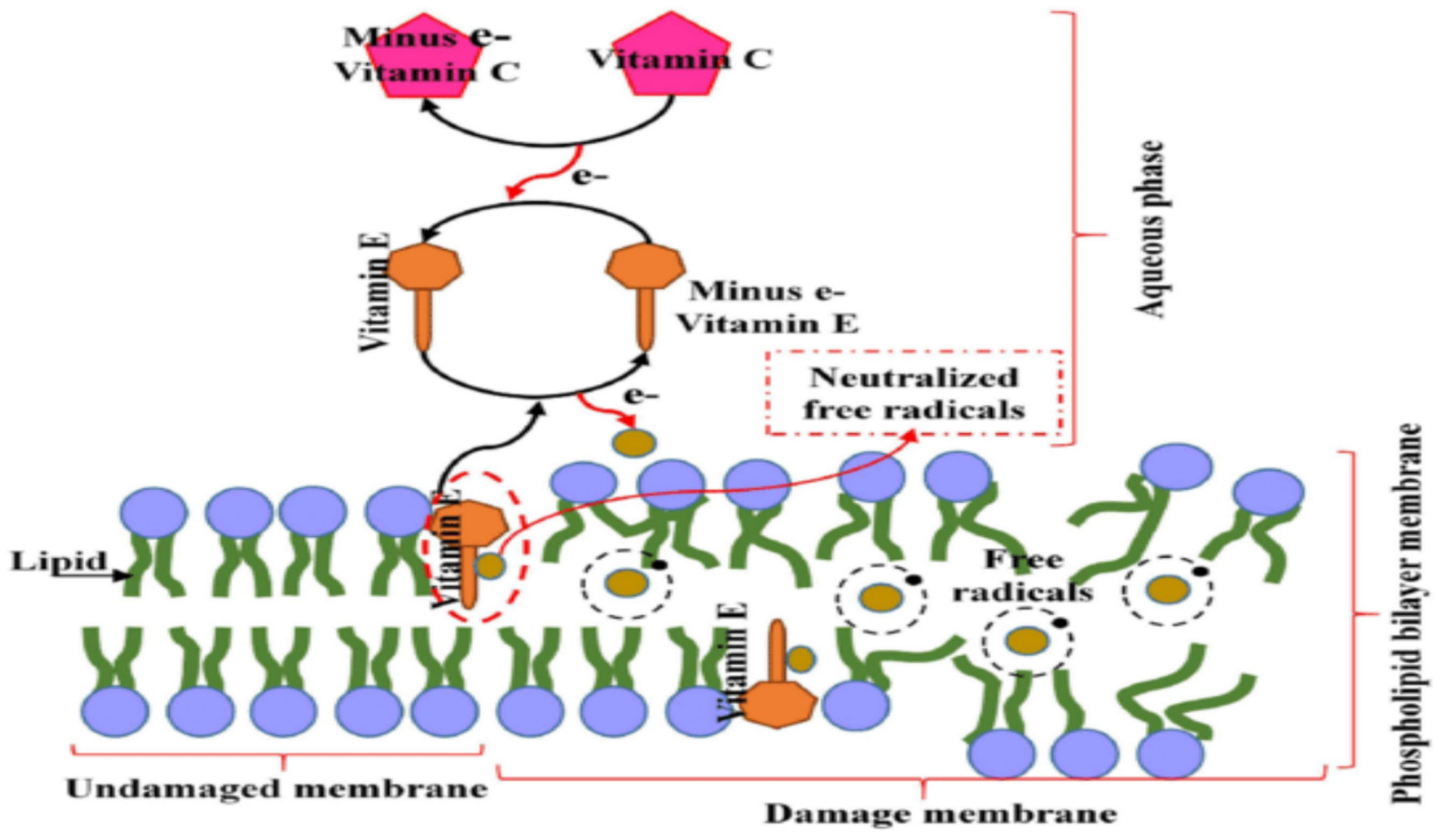
Role of vitamin C in vitamin E Regeneration. Reproduced with permission from (140). The diagram depicts the lipid bilayer membrane structure with its characteristic phospholipid organization, showing both the undamaged membrane (left side) and areas of oxidative damage (right side). The membrane’s hydrophobic core contains embedded vitamin E molecules (shown as yellow structures) strategically positioned to intercept lipid peroxyl radicals and prevent propagation of oxidative chain reactions.

While vitamin E does offer a unique protection against membrane lipid peroxidation and apoptotic signaling in tIRI, its slower pharmacokinetics and potential safety risks limit its use as a stand-alone acute therapy in TT. The most rational translational approach is co-administration with vitamin C, where vitamin C supports rapid aqueous radical scavenging and regenerates α-tocopherol, while vitamin E stabilizes sperm and germ cell membranes during oxidative reperfusion stress (Figure 4).

### 4.3 Previous reports on the synergistic effect of vitamins C and E when co-administered

The synergistic potential of vitamins C and E has been a focus of scientific inquiry for decades and has spanned diverse pathological conditions characterized by oxidative stress (OS), including ischemia–reperfusion injury (IRI) in multiple organs, toxicant-induced testicular damage, and male infertility. Both vitamins have been investigated individually and in combination, with a substantial body of evidence confirming that their co-administration can result in enhanced antioxidant capacity beyond what either can achieve alone (114, 121). This synergy derives from their complementary mechanisms of action, which involve vitamin E neutralizing lipid peroxyl radicals within membranes, while vitamin C recycles oxidized tocopherol back to its reduced, active form (91, 122, 123). Through this redox recycling, vitamin C prevents the accumulation of α-tocopheroxyl radicals and thereby sustains vitamin E’s chain-breaking capacity (115). The “vitamin E-sparing” mechanism is now well-characterized and forms the biochemical basis of their cooperative activity (124, 125).

Experimental work has consistently shown that the combined administration of vitamins C and E efficiently prevents lipid peroxidation (LPO), reduces malondialdehyde (MDA) accumulation, and restores glutathione homeostasis in tissues subjected to IRI (114, 124). In renal IRI, the combined supplementation of these two vitamins improved functional recovery and preserved structural integrity, which outerformed monotherapies (114). Similarly, Layachi and Kechrid (126) reported significant improvements in hepatic glutathione levels with C + E supplementation on cadmium induced oxidative liver injury, showcasing their cooperative ability to restore endogenous antioxidant defenses. These findings are consistent with broader reperfusion models, even in the intestine and kidney, where the C + E pairing attenuated tissue necrosis, improved microvascular perfusion, and suppressed oxidative biomarkers more effectively than either vitamin alone (114, 127, 128).

In reproductive toxicology, several studies have demonstrated that vitamins C and E indeed act synergistically to neutralize testicular toxicity induced by heavy metals and pharmaceuticals. Co-administration of these two vitamins mitigated mercury-induced testicular toxicity by chelating transition metals and suppressing ROS-mediated oxidative damage (27, 35). Similar protective outcomes were observed in lead-exposed rats, where C + E supplementation improved luminal spermatozoa count, seminiferous epithelium integrity, reproductive hormone levels, and overall semen quality (19). The positive impact observed was attributed to vitamin C’s ability to chelate lead, thereby preventing displacement of zinc in zinc-dependent processes, and to vitamin E’s recycling through vitamin C, which in turn helped replenish glutathione and reinforce antioxidant capacity. Likewise, combined supplementation lowered MDA levels and inhibited LPO in vancomycin-induced testicular injury (28), while in models of drug-induced testicular OS, the pairing reduced inflammatory markers, improved serum oxidative balance, and strengthened the blood–testis barrier, underscoring its therapeutic promise in male infertility (129).

Directly within the context of testicular torsion–detorsion, the mechanistic rationale for C + E synergy is particularly compelling. Detorsion induces a surge in ROS that damages lipids, proteins, and DNA, disrupts spermatogenesis, and impairs steroidogenesis. While animal studies have frequently demonstrated the protective effects of either vitamin individually, that is, vitamin E reducing LPO and preserving seminiferous structure (130) and vitamin C attenuating oxidative and histological injury (131, 132), the evidence for co-administration of C and E together in torsion–detorsion models is relatively sparse. Nonetheless, extrapolation from other reperfusion-prone tissues and from male infertility trials suggests that the dual regimen could be particularly effective. In clinical studies on male infertility, daily oral supplementation with vitamin C and vitamin E significantly reduced sperm DNA fragmentation index compared to placebo (133, 134). This outcome is established in *in vitro* studies where semen supplementation with both vitamins reduced ROS-induced DNA damage (135). These data support the hypothesis that their combined use during testicular reperfusion could protect both germ cells and the integrity of testicular steroidogenesis, consistent with prior toxicological and infertility findings (136).

Interestingly, not all studies have been uniformly supportive. Afolabi et al. (137), for instance, observed no reduction in MDA levels in a model of cryptorchidism despite C + E supplementation. They hypothesized that an insufficient concentration of vitamin C relative to vitamin E may have limited the synergistic recycling process, underscoring the importance of dosing ratios in harnessing their combined benefits. That is, while the potential for synergy is strong, optimal timing, dosage, and ratios are essential for realizing maximal benefits (29, 138).

Beyond the testes, the combined antioxidant network of C + E has demonstrated benefits in cardiovascular and visceral IRI. In myocardial reperfusion, for example, peri-procedural administration of both vitamins reduced lipid peroxidation indices and troponin release, with the PREVEC trial explicitly designed on the rationale that vitamin C recycles vitamin E during reperfusion bursts (138, 139). These findings are directly translatable to surgical detorsion, where the timing of reperfusion is predictable and could allow pre-operative administration of the two vitamins. Similarly, intestinal IRI studies have confirmed that hydrocortisone + vitamins C and E outperformed either therapy alone (114), pointing to a general principle: antioxidant synergy is often most effective when aqueous- and lipid-phase antioxidants are paired at the moment of reperfusion.

In essence, the synergy between vitamins C and E is established across various pathological models. This synergistic effect is usually denoted by significant reductions in OS, preservation of tissue structure, and improvement in functional outcomes. In toxicological and infertility contexts, their combination has been associated with improved spermatogenesis, semen parameters, and hormonal balance. While direct torsion–detorsion studies with C + E co-administration do remain relatively limited, there is a strong mechanistic plausibility and proven efficacy already in closely related testicular and systemic IRI models, as well as an established role in male reproductive health. This makes a compelling case for their clinical evaluation in the surgical repair of testicular torsion. The literature suggests that when administered in correct doses and ratios, vitamins C and E can synergistically target ROS, prevent lipid peroxidation, strengthen testicular defense systems, with consequent preservation of fertility potential in the aftermath of ischemia–reperfusion injury.

## 5 Conclusion

Testicular torsion remains a clinical emergency in which surgical detorsion is life-saving for the gonad but paradoxically causes reperfusion injury. Oxidative stress and inflammation are central mediators of this injury, which is denoted by germ cell apoptosis, Leydig/Sertoli dysfunction, and subsequent subfertility. Vitamins C and E can mitigate this OS-induced injury, and they also occupy complementary antioxidant niches, meaning they can act synergistically to better mitigate OS than when used singly. Evidence from experimental torsion–detorsion models, reproductive toxicology, and ischemia–reperfusion studies in other organs shows this synergistic effect, and while clinical translation has not yet been realized, this review highlights that rapid-acting vitamin C in combination with vitamin E (membrane-targeted) is a biologically plausible adjunct to surgical detorsion. We propose that well-designed trials are needed to determine optimal dosing, timing, and long-term fertility outcomes.

## 6 Limitations of the review

A glaring limitation of this review is the fact that the majority of the evidence used is from animal or *in vitro* models. Studies on human testicular torsion using vitamin C and/or E are absent, hence limiting direct clinical extrapolation. Also, since experimental protocols usually vary widely in torsion angle, duration, route, and timing of antioxidant administration, this can make cross-study comparisons difficult. Another important limitation is that most rodents endogenously synthesize vitamin C, unlike humans, which can complicate dose translation. In addition, only a few studies account for the different antioxidant pharmacokinetics, which differ substantially between oral and intravenous preparations.
